# A Single Centre Randomised Control Study to Assess the Impact of Pre-Operative Carbohydrate Loading on Women Undergoing Major Surgery for Epithelial Ovarian Cancer

**DOI:** 10.7759/cureus.10169

**Published:** 2020-08-31

**Authors:** Deniz Al-Hirmizy, Nicholas J Wood, Stanley Ko, Ann Henry, David Nugent, Robert West, Sean Duffy

**Affiliations:** 1 Obstetrics and Gynaecology, Diana Princess of Wales Hospital, Grimsby, GBR; 2 Gynaecologic Oncology, Lancashire Teaching Hospital NHS Foundation Trust, Preston, GBR; 3 School of Medicine and Dentistry, University of Central Lancashire, Preston, GBR; 4 Clinical Oncology/Research and Development Department, Leeds Teaching Hospitals NHS Trust, Leeds, GBR; 5 Gynaecologic Oncology, Leeds Teaching Hospitals NHS Trust, Leeds, GBR; 6 Health Sciences, University of Leeds Institute of Health Sciences, Leeds, GBR; 7 Gynaecology, Leeds Teaching Hospitals NHS Trust, Leeds, GBR

**Keywords:** enhanced recovery after surgery, ovarian cancer, oral carbohydrate loading, length of hospital stay, pain scores, nausea and vomiting scores, post operative care

## Abstract

Objective

Historically, patients have fasted before elective surgery to ensure an empty stomach to avoid aspiration. A fasting-induced catabolic state however may adversely influence recovery after surgery. Our study was designed to test the effect of oral carbohydrate loading on clinical parameters in patients undergoing major surgery for advanced-stage ovarian cancer.

Methods

A double-blinded single-centre randomised trial was designed to recruit 110 patients with advanced-stage epithelial ovarian cancer undergoing either primary surgery, or neoadjuvant chemotherapy prior to debulking surgery. Following written informed consent, the patients were randomised into two groups. Group 1 received the carbohydrate drink (intervention) and group 2 received flavoured water (placebo). The quantity of fluid in both groups was 800ml the night before the surgery and 400ml two hours before the induction of anaesthesia. The primary endpoint of the study was the Length of Hospital Stay (LoHS); the secondary parameters assessed were pain scores, nausea and vomiting scores, bowel function, and postoperative complication rate.

Results

Between March 2009 and December 2011, 80 patients were randomised and 75 completed the study. A decision was made to close the trial early as a change in routine clinical practice meant that patients were admitted on the day of surgery rather than a day before. Analysis of the data revealed that there were no significant differences between the study groups in terms of LoHS and other clinical parameters.

Conclusion

In this single-center study, which failed to recruit the planned number of patients, we were unable to demonstrate that oral carbohydrate intake pre-operatively has significant impact on the recovery process or the length of hospitalisation postoperatively. Future studies should examine all aspects of an Enhanced Recovery Program after Surgery as a package as compared to a single element to enhance patient outcome.

## Introduction

Ovarian cancer is the second commonest gynaecological malignancy after endometrial cancer and the fifth most common female malignancy, with an approximate life time risk of one in 60 [1]. It is the most frequent cause of death among women who develop gynaecological cancer and the incidence is rising [2]. The presenting symptoms of ovarian cancer are often non-specific, which leads to delay between presentation and diagnosis of the disease. The majority of women present with advanced-stage disease and therefore, the five-year survival is relatively low at around 40% [3].

Ovarian cancer predominantly presents in postmenopausal women with a median age of diagnosis of 63 years. In the UK, 7000 new cases of epithelial ovarian cancer are diagnosed every year, with 4200 women dying from the disease annually [4].

As patients are often undergoing treatment at an advanced age, many of them may already be experiencing other medical challenges and co-morbidities. This increases the burden on their health and can adversely affect their treatment options and disease progression [5].

Surgery is often the mainstay in the management of ovarian cancer. It can be undertaken as primary treatment followed by postoperative chemotherapy or alternatively, it can be in the form of interval debulking surgery (IDS), defined as surgery performed after an initial course of chemotherapy [6].

Nearly 4% of the population undergo surgery every year globally [7]. Surgery and trauma can cause major metabolic and hormonal changes in the body including an increase in insulin resistance which adversely affects the recovery of the patient postoperatively [8]. Over the last decade, Enhanced Recovery Programs after Surgery (ERAS) have been introduced to improve surgical recovery and outcomes.

ERAS programmes have a multimodal approach that includes pre, intra-, and postoperative interventions with the main aim to reduce the stressful effect of surgery and to speed up patient’s recovery [9]. Its effectiveness has been proven in numerous studies and clinical trials [10]. The key elements of ERAS include: A) extensive preoperative patient education involving patient objectives and anticipations about surgery; B) pronounced reduction of preoperative fasting time and the utilisation of preoperative oral carbohydrate before surgery; C) multimodal pain control approach commencing with non-opioid analgesia up to regional anaesthesia with the goal of reducing the opioid use; D) rapid recommencement of normal diet as well as encouragement of early mobilisation after surgery.

ERAS programmes have shown improved results in terms of reduced hospital stay and enhanced surgical recovery [11]. Since 2006, the use of ERAS has been developed in the field of colorectal surgery [12] and became well established with the development of consensus and guidelines in 2011 [13]. Following that, a subsequent move has been initiated towards implementing the programme into other surgical fields, including gynaecology and gynaecological oncology [14,15].

We conducted a single-centre two-arm randomised controlled trial to test the effect of one aspect of ERAS, preoperative oral carbohydrate loading, in patients with advanced-stage epithelial ovarian cancer undergoing surgery. The primary outcome of the study was the length of hospital stay. We also investigated secondary clinical parameters such as pain score, nausea and vomiting score, bowel function, and postoperative surgical complication rate. The recruitment for our study commenced in March 2009 before the routine introduction of ERAS into clinical practice in the gynaecological oncology field.

## Materials and methods

Patient population

Ethical approval for the study was granted by the Leeds East Regional Ethics Committee in October 2008. Potential participants were identified from the weekly Leeds Gynaecological Cancer Centre Specialist Multi-Disciplinary Team meetings (MDT). The study inclusion criteria included patients with presumed stage III/IV epithelial ovarian cancer scheduled for major pelvic surgery, who were willing and able to give written informed consent. Both chemotherapy naïve and post-chemotherapy patients were approached. Exclusion criteria included 1) diabetes mellitus (type I or type II), 2) any pre-morbid disorder of gastric emptying, 3) morbid obesity (BMI of >50) and/or 4) citrus allergies. Patients were admitted the day before surgery and on admission approached by the researcher, who provided both verbal and written information about the study.

Randomisation

A double-blinded single-centre randomised trial was designed to recruit 110 patients with advanced-stage epithelial ovarian cancer undergoing either primary surgery or neoadjuvant chemotherapy prior to surgery for advanced-stage epithelial ovarian cancer.

On admission and following written consent, participants were randomised into two groups: routine care (placebo/flavoured water) or pre-operative oral carbohydrate loading (intervention/CHO) by using sequentially numbered opaque, sealed envelopes containing cards marked “f. water” (flavoured water) or “pre-Op” (oral carbohydrate/CHO; Nutricia Clinical Care, Wiltshire, UK). Random number allocation was used to generate 110 sealed opaque envelopes with allocation to either placebo or intervention (55 cards in each way). These cards were kept with the store of product of (pre-Op™) and flavoured water on the gynaecological oncology ward. The clinical fellow (D Al-H) was responsible for drawing sequential envelopes the night prior to surgery. Then the clinical fellow would hand the sealed envelope to one of the nursing staff who was not directly involved in the study to open it and to decant the assigned drink (pre-Op™ or f. water).

Intervention

**Figure 1:**
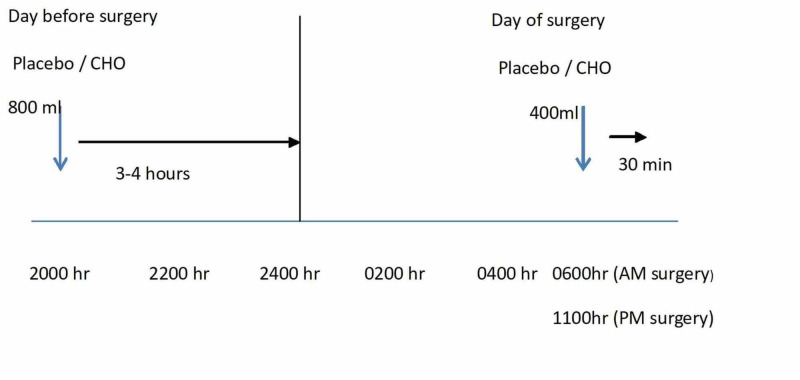
Schema of timings and volumes of oral administration of pre-operative carbohydrates and placebo CHO: carbohydrate

Following randomisation, the appropriate substance (flavoured water or pre-operative oral carbohydrate loading) was decanted into two sterile containers for evening and morning administration by the nursing staff. These were labelled with the patient’s name and kept refrigerated on the ward until administration at the prescribed time. By performing this method, we aimed to ensure “blinding” of the surgical team. The evening drink was commenced at 20.00 hours while the morning one was given at 6.00 am for morning surgery and 11.00 am for afternoon surgery (Figure 1). Both drinks looked exactly the same (colourless) and tasted closely similar. The pre-Op™ drink is a food for special medical purpose administered under medical supervision, designed specifically to switch patients from a fasted to a fed state prior to surgery. It contains 12.6g/100mls of carbohydrate (polysaccharides, sugars, and lactose). The duration of consumption of the evening drink was made to be three to four hours due to the larger quantity of the drink (800mls), while the duration of the morning drink was 30 minutes.

Data collection

Background clinical information was collected about previous history of motion sickness, history of sickness associated with general anaesthesia, smoking history, and any other issues with nausea or vomiting.

Participants were asked to complete a pain score by using the descriptive visual analogue scale (VAS). This is a validated tool and one of the most frequently used methods in rating pain scales. It is easy to use, requires no verbal or reading skills, and can be used in a variety of settings. The pain scores from 24 hours before admission up to three days postoperatively were collected daily.

The use of any analgesia or anti-emetics in the 24 hours prior to admission was recorded. The amount of oral fluid consumed was recorded from 18.00 hours the day before surgery.

Subsequently from day 0 to three, daily information of pain scores, nausea and vomiting, analgesia and anti-emetics requirements, and information on passing first flatus and bowel motion were obtained directly by asking the patients and confirmed from the nursing staff and the clinical notes. We used a validated tool, Rhodes Index, for recording nausea and vomiting scores [16]. It scores, as categorical variables, the number of vomiting episodes per day, the volume of the vomit, the degree and length of nausea and retching, as well as the distress associated with the condition. We used the above assessment form to record our nausea and vomiting data. Post-surgical complications were also recorded.

In addition, Short Form-8 (SF-8) questionnaire to assess quality of life (QoL) of the patients, which is a generic health questionnaire capture reliable and scientifically valid patient-reported health outcome information, was collected on admission to hospital and on day three postoperatively. This form has been adapted to assess female cancer patient population [17].

The Physiological and Operative Severity Score for the enumeration of Mortality and morbidity (POSSUM) score is a predictive score of morbidity and mortality that has been validated for general, orthopaedic and cardiothoracic surgery. It has been assessed in gynaecological oncology surgery and found to consistently overestimate morbidity and mortality in a UK centre [18]. It is, however, a complex and robust method of comparing morbidity between patient populations and therefore appropriate to be used to compare the study and control group.

The POSSUM score consists of two parts: physiological, which scores between 12-88 and includes preoperative investigation results such as vital signs (blood pressure measurement [systolic mmHg], pulse rate and Glasgow coma scale); cardiac sign; electrocardiogram (ECG); chest x-ray; respiratory symptoms; and blood test results (haemoglobin [g/100ml], white cell count [x10⁹/L], blood urea [mmol/L], blood sodium [mmol/L] and potassium [mmol/L]); in addition to the above, the physiological part scores the age of the patient as well. The second part is the operative POSSUM score which ranges between 6-48 and involves scoring for intra-operative features like severity of the operation score (minor, moderate, major, or major plus); single or multiple procedures (1, 2, and >2); total volume of blood loss (ml) (<100, 101-500, 501-999 and >999); peritoneal contamination during the operation (none, minor with serous fluid, local pus and free bowel content of pus or blood); presence of malignancy (none, primary only, nodal metastases, and distal metastases); and mode of operation (elective or emergency ). All the participants in the trial had elective surgery for advanced-stage ovarian malignancy. The lower the scores the fitter the patients and the better outcomes.

Statistical analysis and sample size determination

The sample size calculation was based upon data gathered from a historic sample of 46 patients with stage III/IV epithelial ovarian cancer undergoing primary surgery for the period 2006-2007 at St James’s University Hospital in Leeds. The primary outcome measure was Length of Hospital Stay (LoHS). This was seen to have a skew distribution but on taking a logarithmic transformation, a very close approximation to normality was seen. The statistical analysis of the above historic sample revealed that the mean LoHS was 7.24 days, mean of log LoHS was 1.98, and the standard deviation of log LoHS was 0.44. It was considered that a difference in LoHS of two days is clinically significant and so the smallest difference to be detected in log LoHS is log (9.24) - log (7.24) = 0.24.

Subsequent power calculation for the primary endpoint of the study (LoHS) suggested 106 patients would provide 80% power (p-value of 0.05) to demonstrate a clinically significant reduction in mean LoHS of two days. To allow for non-compliance the planned sample size was 110 participants.

The main purpose of the trial was to test the hypothesis that oral carbohydrate drink changes the LoHS after surgery. Therefore, the null hypothesis of the study was that oral carbohydrate loading does not change the length of hospital stay after surgery. The alternative hypothesis states that the oral carbohydrate administration pre-operatively does shorten or lengthen the hospital stay after surgery.

Since LoHS is known to have a skewed distribution, the natural logarithm of LoHS was calculated and used as the primary outcome. The transformation produces a distribution much closer to the normal distribution.

The original analysis plan was to compare the means of log LoHS between the CHO and placebo groups using a two-sample t-test. Lack of balance post-randomisation meant that the analysis plan was changed to employ a linear regression model.

We explored reasons for variation in log LoHS, and therefore LoHS by regression. This enabled the adjustment of results for factors that may not have been sufficiently balanced due to randomisation. During regression modelling, factors that were not significant at the 5% level for a Wald test (a t-test for parameter coefficients) were dropped.

We had two types of variables: categorical variables, which contain a finite number of categories or distinct groups and might not be ordered (e.g. type of surgery, pain score, smoking status, and complication rate), and continuous variables, which are defined as numeric variables that have a large range of values (e.g. age, BMI, and POSSUM score).

Before commencing regression modelling, direct (unadjusted) comparisons were made between the two groups, for this we have applied t-tests for the continuous data and chi-squire tests (x²) for the categorical data.

A statistical package of R version 3.5.1 (2018-07-02) was utilised to analyse the data.

## Results

**Figure 2:**
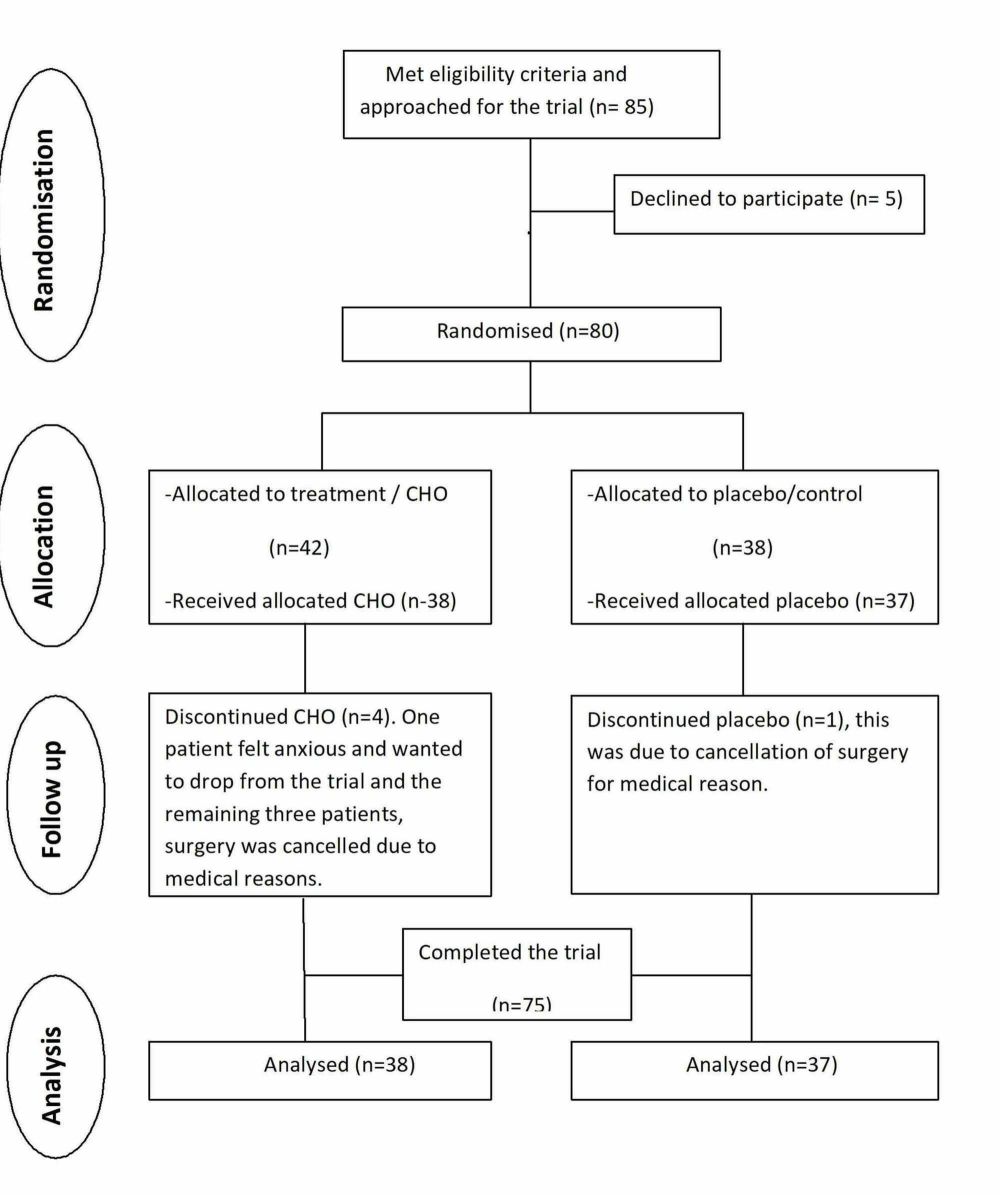
CONSORT flow diagram of the progress though the phases of a parallel randomised trial. CHO=carbohydrate, CONSORT=Consolidating Standards Of Reporting Trials

A total of 85 patients were approached between March 2009 and December 2011. Five patients declined participation in the study; the remaining eighty patients consented and were randomised into carbohydrate/intervention or placebo/control groups. Four patients had surgery cancelled for medical reasons and one patient withdrew her consent preoperatively and prior to any collection of data on the day of surgery. A total of 75 patients were available for analysis. The overall summary is shown in Figure 2 using the CONSORT (Consolidating Standards Of Reporting Trials) flow diagram.

The study was initially designed to recruit 110 patients with advanced stage ovarian cancer undergoing surgery. However, it was terminated early because of changes in hospital admission practice. From the end of 2011 patients were admitted on the day of the operation and therefore the study randomisation and intervention procedure was no longer viable.

Compliance

**Figure 3:**
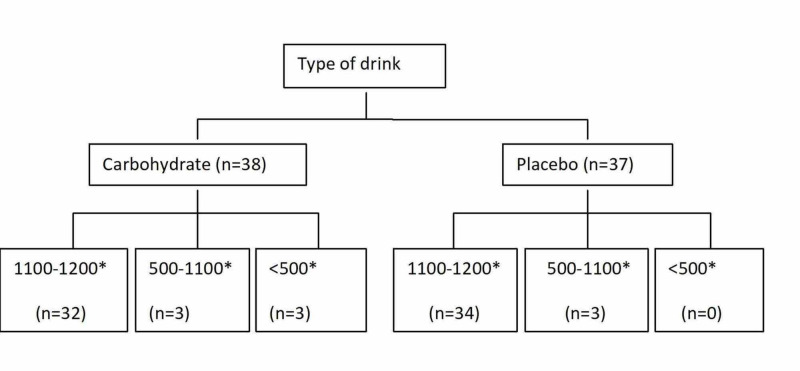
Number of patients in each group with various amounts of study drinks. *= the amount represented by millilitres (ml).

Compliance with the intervention was excellent with the majority (88%) of the study population having the full or nearly full amount (1100-1200) of the study drink (either carbohydrate or placebo). The remaining 8% and 4% of the study groups had 500-1100 ml and <500 ml, respectively (Figure 3).

Patient characteristics

The two groups were comparable in respect to age and BMI. The mean age for the carbohydrate loading group was 63.2 (range 44-85) years and for the placebo group was 61 (range 38-83) years. The BMI was 27.1 (21-38) and 26.4 (19-40.8), respectively. The number of smokers was also comparable in both groups (CHO group 13.2%, placebo 16.2%).

In regards to the type of surgery, i.e. primary or interval debulking surgery (IDS), the percentage of patients who had primary surgery was 42.7% and of those 62.5% had received carbohydrate loading and 37.5% had received placebo. For the remaining 57.3% who had IDS, 41.8% had received carbohydrate and 58.2% had taken placebo. To assess whether this difference in proportions undergoing primary surgery or IDS may have impacted on recovery, we undertook a post-hoc analysis using the POSSUM score.

The POSSUM physiological score was similar in both study groups with mean score in the CHO group of 17.7 (±3.42) and in the placebo group of 17.2 (±3.32) (p=0.622). However, when we compared the operative POSSUM score between the two study groups, we found that the score was significantly lower in the placebo group with mean score of 14.49 (±2.64) versus 16.97 (±5.76) in CHO group (p=0.019). This suggests that overall the patients in the placebo group had less extensive surgery than those in the carbohydrate group. A likely explanation was that there was a higher number of IDS in the placebo group (67.6%) compared to just under half of the carbohydrate loading group (47.4%).

No significant differences were found between the two study groups in regards to baseline information on history of motion sickness (p=0.051) nor sickness related with general anaesthesia (p=0.560).

No significant differences were found between the two groups in regards to the pain killers in the 24 hours prior to hospital admission (p=0.892) and anti-emetics medication taken up to one day before hospital admission (p=0.978).

Comparable findings were also identified between the two groups for the characteristics of bowel resection during surgery (p=0.371), stoma formation (p=0.627) and amount of residual disease following surgery (p=0.996) (Table 1).

**Table 1:** Patient characteristics in both study groups. Statistically significant values are in bold. IDS*=Interval Debulking Surgery, GA*= General Anaesthesia, POSSUM=Physiological and Operative Severity Score for the enumeration of Mortality and morbidity

Characteristics	Carbohydrate (n=38)	Placebo (n=37)	P value
Age mean (range) years	63.2 (44-85)	61 (38-83)	0.386
BMI mean (range)	27.1 (21-38)	26.4 (19-40.8)	0.621
Smoker %	(n=5) 13.2%	(n=6) 16.2%	0.962
Non-smoker%	(n=33) 86.8%	(n=31) 83.8%	
Type of surgery% IDS* (n=43)	(n=18) 47.4%	(n=25) 67.6%	0.125
Primary surgery (n=32)	(n=20) 52.6%	(n=12) 32.4%	
Motion sickness %	None (n=32) 86.5% Mild (n=1) 2.7% Moderate (n=2) 5.4% Severe (n=2) 5.4%	None (n=26) 70.3% Mild (n=7) 18.9% Moderate (n=4) 10.8% Severe (n=0) 0%	0.051
Sickness with GA*%	No (n=27) 84.4% Yes (n=5) 15.6%	No (n=21) 75% Yes (n=7) 25%	0.560
Pain killer in 24 hours %	No (n=22) 57.9% Yes (n=16) 42.1%	No (n=22) 59.5% Yes (n=15) 40.5%	0.892
Anti-emetic in 24 hours %	No (n=36) 94.7% Yes (n=2) 5.3%	No (n=35) 94.6% Yes (n=2) 5.4%	0.978
POSSUM Physiological score (mean(SD)) (pre-operative)	17.7(3.42)	17.2(3.32)	0.622
POSSUM operative score (mean(SD)) (intra-operative)	16.97(5.76)	14.49(2.64)	0.019
Bowel resection (%)	No (n=34) 89.5% Yes (n=4) 10.5%	No (n=36) 97.3% Yes (n=1) 2.7%	0.371
Stoma (%)	No (n=35) 92.1% Yes (n=3) 7.9%	No (n=36) 97.3% Yes (n=1) 2.7%	0.627
Disease residual (0-5) (mean(SD))	0.95(1.18)	0.95(1.15)	0.996

Clinical parameters

The primary outcome for the trial was Length of Hospital Stay (LoHS). From Table 2 the unadjusted analysis showed a statistically significant difference between the two groups: the median duration being longer at six days for the CHO/ intervention group and five days for the placebo/control group. It has also been observed that despite randomisation, the mean operative POSSUM scores of the two groups were unbalanced, so that overall there was more extensive surgery in the intervention/CHO group. Consequently, we do not consider the unadjusted comparison to be a fair one.

**Table 2:** Clinical parameters in both study groups. Statistically significant values are in bold.

Study parameters	Carbohydrate (n=38)	Placebo (n=37)	Pvalue
Primary end point Length of Hospital Stay (LoHS) median (days)	6	5	0.014
(Mean(SD))	6.47(2.84)	5.22(1.08)	
Secondary outcomes			
Pain score (mean(SD)) Pre-operatively( Day 0)	0.21(0.53)	0.14(0.42)	0.496
Day 1	1.74(0.92)	1.68(0.75)	0.753
Day 2	1.61(0.59)	1.59(0.72)	0.945
Day 3	1.16(0.68)	1.11(0.52)	0.742
Additional analgesia (mean(SD)) Day 0	1.11(1.39)	1.24(1.55)	0.686
Analgesia requirement (mean(SD)) Day 1	2.76(2.54)	3.19(2.89)	0.500
Day 2	7.45(2.82)	7.03(2.62)	0.506
Day3	6.26(2.49)	5.75(2.93)	0.419
Type of analgesia peri-operatively % (nil analgesia)	(n=1) 2.6%	(n=3) 8.1%	0.658
Continuous epidural	(n=10) 26.3%	(n=11) 29.7%	
Continuous epidural + patient control analgesia (PCA)	(n=2) 5.2%	(n=4) 10.8%	
patient control analgesia	(n=19) 50%	(n=13) 35.1%	
Single shot spinal	(n=5) 13.2%	(n=4) 10.8%	
Single shot spinal + patient control analgesia (PCA)	(n=1) 2.6%	(n=2) 5.4%	
Nausea & vomiting score(mean(SD)) Day0	0.04(0.15)	0.02(0.12)	0.623
Day 1	0.49(0.72)	0.27(0.36)	0.089
Day2	0.26(0.36)	0.16(0.40)	0.289
Day3	0.50(0.81)	0.16(0.46)	0.030
Anti-emetic requirement (mean) Day1	0.74(1.00)	0.70(0.91)	0.878
Day2	0.87(1.09)	0.65(1.03)	0.374
Day3	0.79(0.99)	0.36(0.80)	0.045
Bowel function Passing first flatus (days) (median) (mean(SD))	2 2.89(1.80)	3 2.68(1.06)	0.523
Bowel motion (days)(median) (mean(SD))	4.5 4.78(2.98)	4 3.71(1.33)	0.225
Complication rate (%)	No (n=24) 63.2% Yes(n=14) 36.8%	No (n=27) 73.0% Yes (n=10) 27.0%	0.507
Quality of Life (QoL) questionnaire (mean(SD)) Admission day	26.35(5.49)	26.17(5.43)	0.689
Day 3	18.45(6.31)	17.62(6.69)	

To provide a fairer comparison and adjust for relevant factors that may impact LoHS, we fitted a regression model. This regressed the primary outcome, LoHS, on factors (independent variables) relevant to patients up to and including surgery. These factors may have influenced recovery and therefore LoHS. Specifically, the factors were: type of surgery (IDS/primary surgery), complication status, physiological POSSUM score, operative POSSUM score, age, and BMI. The type of drink (CHO/placebo) was also included as the factor of interest.

The result of the model fitting (Table 3) shows that the operative POSSUM score was strongly associated with LoHS with each point of the score being associated with an increase in LoHS of 0.292 days (i.e. seven hours). Also, complications were associated with an increased stay of 0.917 days (i.e. 22 hours). There was little evidence that any other factor was associated with LoHS since no other factor was statistically significant according to the Wald test (the t test for the coefficient).

**Table 3:** Table of coefficients for the regression of Length of Hospital Stay (LoHS). Statistically significant values are in bold. IDS=Interval Debulking Surgery, POSSUM=Physiological and Operative Severity Score for the enumeration of Mortality and morbidity, CHO=carbohydrate

(Intercept)	Estimate	Standardized	Error	Significance
Constant	-1.07377	1.65199	-0.650	0.517
drink (CHO)	0.30760	0.38648	0.796	0.428
POSSUM (Op)	0.29240	0.04154	7.040	0.000
IDS.Primary	0.38981	0.40423	0.964	0.338
POSSUM (Physio)	0.03362	0.05608	0.600	0.550
Complications	0.91695	0.31660	2.896	0.005
Age	0.01466	0.01762	0.832	0.408
BMI	0.01072	0.03278	0.327	0.744

These findings are consistent with clinical experience that patients require longer recovery after more extensive surgery and that complications can increase LoHS. Note that after adjustment for these factors, the type of drink (CHO/placebo) was no longer statistically significant (Wald test p=0.428). Importantly the lack of significance in the regression model showed that there was little evidence that the type of drink was associated with LoHS.It is useful to consider the relative contributions of the factors identified by the regression model and the type of drink. From Table 1, the mean operative POSSUM scores differed by 2.5 points between the two trial arms (CHO=16.97 and placebo=14.49). This represents an anticipated difference in LoHS of (2.5*7.4 hours=18.5 hours).

The difference in complication rates between the two study arms (Table 2) was 10%, representing a difference in anticipated LoHS of 10% * 22 hours=2.2 hours. These therefore contribute a substantial proportion of the observed difference in LoHS between the two groups (six days versus five days) (Table 2).

**Figure 4:**
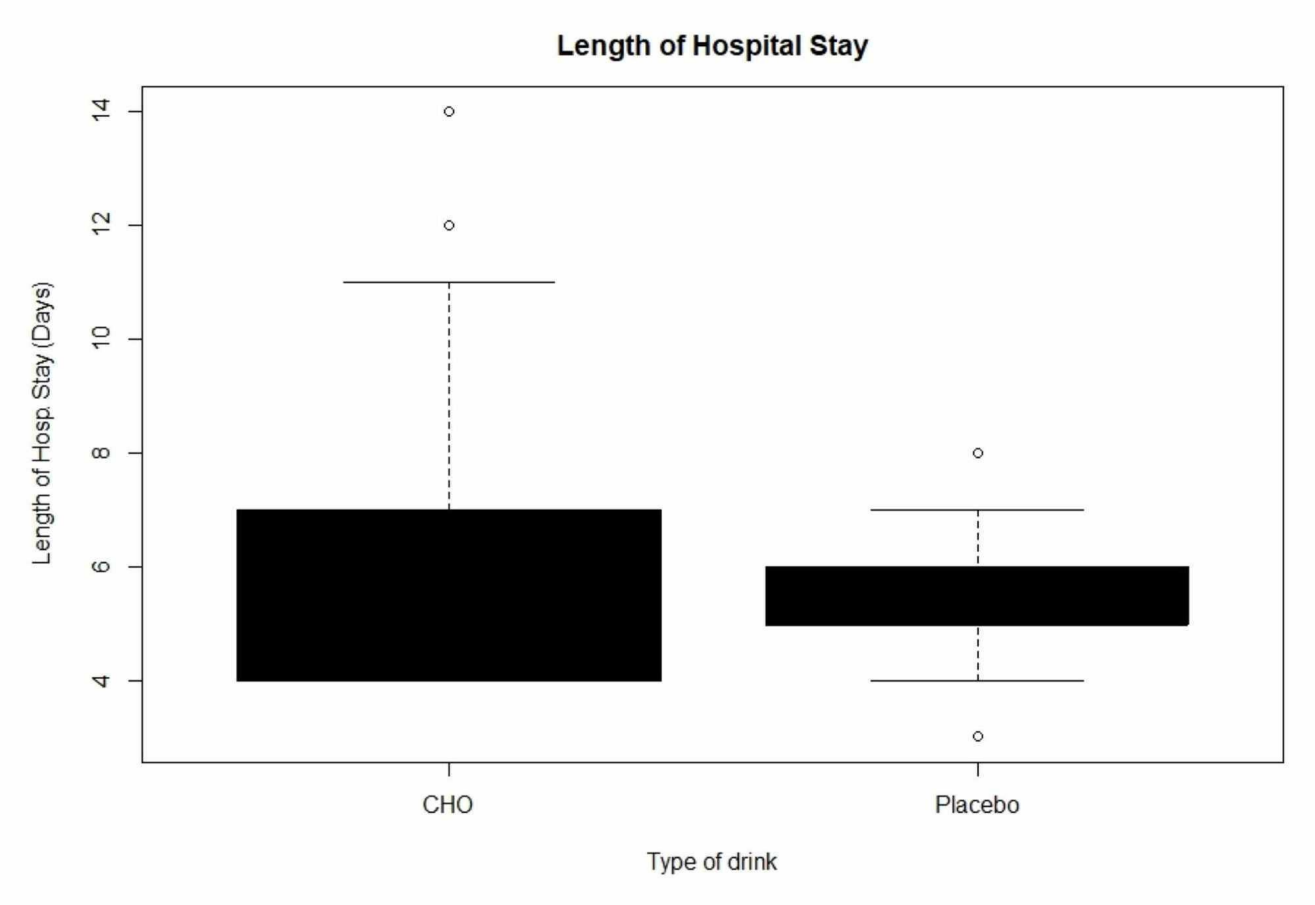
Length of Hospital Stay (LoHS) between the two study groups.

The following Box plot (Figure 4) shows the differences in length of hospital stay in patients from both study drinks.

Comparison of the secondary endpoints of the study in regards to pain scores, analgesia requirements, nausea and vomiting scores, anti-emetic requirements, resumption of bowel function and generic heath questionnaire showed no significant differences between the two study groups.

## Discussion

In this single-centre study, which failed to recruit the planned number of patients, we were unable to demonstrate that oral carbohydrate intake pre-operatively has significant impact on the recovery process or the length of hospitalisation postoperatively in patients undergoing pelvic surgery for ovarian cancer compared with oral hydration alone. The study failed to recruit the planned sample size and was closed early due to a change in routine clinical care that made it impractical to continue. Changes in routine admission policy, i.e. move to patients being admitted on the day of surgery instead of day before, made it extremely difficult to administer the study drink and ensure the blindness of the study which led to early termination of patient recruitment.

On analysing our results, we were unable to determine that oral carbohydrate loading pre-operatively has significant impact on the patient’s length of hospital stay postoperatively. Our initial analysis showed that oral CHO loading increased the length of stay by one day more than the placebo drink but we believe that the explanation for this is that the groups were unbalanced despite randomisation. This was supported by post-hoc data analysis of POSSUM scores.

Analysis of patient characteristics shows that more patients had IDS in the placebo arm as compared to intervention (carbohydrate) arm. A number of studies demonstrate that IDS results in shorter hospital stay than PDS [19]. Since two-thirds of our IDS population had placebo, and since our observational analysis had revealed that the IDS group of patients do better in terms of recovery than the PDS group regardless of the type of intervention (placebo or carbohydrate drinks),we infer it is the type of surgery rather than the type of drink that made the LoHS shorter in the placebo group.

Evidence including randomised control trials comparing interval debulking surgery (IDS) to primary debulking surgery in advanced-stage ovarian cancer (i.e. stage III, IV) has also shown less operative morbidity and mortality with similar long term overall survival in the former group. Over time IDS gained more popularity and started to become the preferred management approach during the process of recruitment to the study, but this was not the case when the trial was initially designed [20].

Another factor that was found to be significantly different between the two study groups is operative POSSUM score. The higher the score of the operative POSSUM the more extensive surgery and the more time would be needed for the recovery of patient postoperatively which eventually leads to longer time of hospital stay. Our study showed that the mean (±SD) for operative POSSUM score in the CHO group was 16.97 (±5.76) versus 14.49 (±2.64) in the placebo group (p=0.019).

To determine the association between the operative POSSUM score and type of surgery (IDS, PDS), we carried out a post-hoc analysis to compare operative POSSUM score between IDS and PDS regardless the type of the study drink (CHO, placebo). We found that the IDS group had lower operative POSSUM scores with mean (±SD) score of 15.67 (±5.21) versus 15.84 (±3.80) in PDS. This was not statistically significant, but suggested a trend that IDS group do better postoperatively.

In addition, we looked at further historic data retrospectively (2006-2016). These data were obtained from St James's University Hospital Gynaecological Oncology department in Leeds. We found that the mean and median LoHS improved over time. This could be due to improving care using some elements of ERAS programme (e.g. no bowel preparation before surgery, early mobilisation postoperatively, early feeding, and early removal of catheter post-surgery). This makes it difficult to demonstrate pre-operative oral CHO intake does not significantly reduce the length of hospitalisation as it has already reduced it over the years.

A literature search has revealed that few systematic reviews have addressed the current evidence for ERAS in Gynaecological Oncology [21-24]. These reviews were based on non-randomised studies and contained high risk of bias and very few studies looked at individual ERAS elements.

Lindemann et at. in their 2017 systematic review had identified studies that evaluated individual components of ERAS including early feeding, perioperative fluid management, postoperative analgesia, avoidance of drain and nasogastric decompression and postoperative chewing gum. None of these studies were randomised controlled trials in patients with ovarian cancer.

Reduction of fasting time pre-operatively by administrating carbohydrate loading has been part of some of the published ERAS programme. Its beneficial effect in reducing fasting time is uncertain [25]. In this Cochrane review carbohydrate loading as an individual component of the ERAS was addressed, however, their inclusion criteria were different than our study. They included any type of elective surgery not specifically ovarian cancer patients, which was one of the fundamental criteria in our study. Furthermore, their intervention groups have involved patients receiving oral or parental carbohydrate loading compared with our study which addressed oral administration only.

Our study shows patient acceptance of the non-fasting approaches preoperatively and excellent compliance with either of the study drinks in respect to their amount, palatability and taste; both were considered satisfactory with no negative feedback. Evidence shows the safety of clear fluid intake up to two hours before surgery [26], avoiding long hours of fasting leading to increase insulin résistance which has an adverse effect on postoperative recovery and increases complication rates post-surgery. A number of studies including systemic reviews and meta-analyses have shown that pre-operative oral carbohydrate can antagonise the harmful effect of incremental insulin resistance [13,25,27]. Studies have also shown that adapting a liberal approach of hydrating patients prior to their surgery improves their psychological well-being, specifically reducing anxiety and thirst [28,29].

## Conclusions

The trial was powered for the primary outcome of LoHS. The results show that there is little evidence to support that oral CHO loading pre-operatively may reduce the length of hospital stay. However, there were some confounding factors in our study, specifically unbalanced type of surgery (IDS, PDS) between the two groups, failure to recruit the total number of patients for the trial, and general trend over the study period for shorter hospitals stays. Also testing oral CHO drink as a single element of ERAS programme, rather than the full package of fast-track recovery programme, may limit the potential benefits that could be achieved. All of these factors are limitations to our study. One of our study population characteristics that we looked at was operative POSSUM score, where we found that the mean score of the operative POSSUM was significantly higher in the CHO group compared to the placebo group. This indicated that more complicated surgery was performed in the CHO group than the placebo which could be explained by the increased number of IDS in placebo arm compared to CHO arm (almost two-thirds of IDS in placebo while less than half of IDS in CHO group).

Further research including consensus and randomised control trials are needed in this field to gain better understanding of the effect of ERAS programme in Gynaecological Oncology patients. Future studies should examine all aspects of ERAS programme as a package as compared to a single element to enhance patient outcome.
